# Global prevalence of claudin 18 isoform 2 in tumors of patients with locally advanced unresectable or metastatic gastric or gastroesophageal junction adenocarcinoma

**DOI:** 10.1007/s10120-024-01518-1

**Published:** 2024-07-02

**Authors:** Kohei Shitara, Rui-Hua Xu, Jaffer A. Ajani, Diarmuid Moran, Abraham Guerrero, Ran Li, Janet Pavese, Maria Matsangou, Pranob Bhattacharya, Yoko Ueno, Xuewei Wang, Manish A. Shah

**Affiliations:** 1 Department of Gastrointestinal Oncology, National Cancer Center Hospital East, Kashiwa City, Chiba Japan; 2 Department of Medical Oncology, Sun Yat-Sen University Cancer Center, State Key Laboratory of Oncology in South China, Collaborative Innovation Center for Cancer Medicine, Guangzhou, China; 3 Department of Gastrointestinal Medical Oncology, Division of Cancer Medicine, The University of Texas, MD Anderson Cancer Center, Houston, TX USA; 4 Astellas Pharma Global Development, Inc., Northbrook, IL USA; 5 Astellas Pharma Inc., Tokyo, Japan; 6 Division of Hematology and Medical Oncology, Weill Cornell Medical College, New York City, NY USA

**Keywords:** Biomarkers, Gastric cancer, Gastroesophageal cancer, Claudin 18 isoform 2 prevalence, Zolbetuximab

## Abstract

**Background:**

Limited data exist for global prevalence of claudin 18 isoform 2 (CLDN18.2) positivity and association of CLDN18.2 status with clinical and tumor characteristics in patients with locally advanced (LA) unresectable or metastatic gastric or gastroesophageal junction (mG/GEJ) adenocarcinoma. We report prevalence of CLDN18.2 positivity (phase 3; SPOTLIGHT, NCT03504397; GLOW, NCT03653507) and concordance of CLDN18.2 status between a subset of pair-matched tumor samples (phase 2, ILUSTRO, NCT03505320; phase 1, NCT03528629) from clinical studies of zolbetuximab.

**Methods:**

Tumor samples from patients with LA unresectable or mG/GEJ adenocarcinoma were tested for CLDN18.2 status by immunohistochemistry. Human epidermal growth factor receptor 2 (HER2) expression was tested per central or local assessment.

**Results:**

Across SPOTLIGHT and GLOW, the prevalence of CLDN18.2 positivity (≥ 75% of tumor cells demonstrating moderate-to-strong membranous CLDN18 staining) was 38.4%. Prevalence was similar in gastric versus GEJ adenocarcinoma samples and regardless of collection method (biopsy versus resection) or collection site (primary versus metastatic). CLDN18.2 positivity was most prevalent in patients with diffuse-type tumors. In ILUSTRO and the phase 1 study, concordance of CLDN18.2 positivity was 61.1% between archival (i.e., any time before treatment) and baseline (i.e., ≤ 3 months before first treatment) samples, and concordance of any CLDN18 staining (≥ 1% of tumor cells demonstrating moderate-to-strong membranous CLDN18 staining) was 88.9%.

**Conclusions:**

CLDN18.2 was a highly prevalent biomarker in patients with HER2-negative, LA unresectable or mG/GEJ adenocarcinoma. CLDN18.2 positivity remained relatively stable over time in many patients. Biomarker testing for CLDN18.2 should be considered in standard clinical practice in these patients.

**Supplementary Information:**

The online version contains supplementary material available at 10.1007/s10120-024-01518-1.

## Introduction

Gastric and gastroesophageal junction (G/GEJ) adenocarcinoma are among the leading causes of cancer-related deaths worldwide [1]. Standard first-line (1L) treatment for patients with locally advanced (LA) unresectable or metastatic G/GEJ (mG/GEJ) adenocarcinoma includes fluoropyrimidine- and platinum-based chemotherapy regimens, including modified FOLFOX (mFOLFOX6; folinic acid, fluorouracil, and oxaliplatin) and CAPOX (capecitabine and oxaliplatin); patients on these regimens have a median overall survival (OS) of about 1 year [2–6]. The combination of chemotherapy with targeted therapies and immunotherapies has improved survival in some patients [7]. Trastuzumab (anti-human epidermal growth factor receptor 2 [HER2]) is approved for the treatment of patients with HER2-positive tumors [3–6, 8]. Nivolumab (anti-programmed cell death 1 receptor [PD-1]) is approved in several countries for the treatment of patients with HER2-negative tumors, though efficacy has been demonstrated mainly in patients with tumors with a programmed cell death ligand 1 (PD-L1) combined positive score (CPS) ≥ 5 [3–6, 9, 10]. Survival rates remain low; additional biomarker-targeted therapies are needed to improve survival in patients with HER2-negative, LA unresectable or mG/GEJ adenocarcinoma [11].

Claudin 18 isoform 2 (CLDN18.2) is a tight junction protein expressed on normal gastric epithelial cells that is retained during malignant transformation [12–19]. CLDN18.2 is the dominant claudin 18 (CLDN18) isoform expressed in both normal and malignant gastric epithelial cells and is also frequently expressed in tumors derived from organs that normally do not express CLDN18.2, including pancreatic and esophageal adenocarcinomas [12, 20–23]. As malignant cells lose polarization, CLDN18.2 may become exposed on the surface of G/GEJ adenocarcinoma cells, which may make it available to targeting with monoclonal antibodies [13–18, 24].

Zolbetuximab is a chimeric immunoglobulin G1 monoclonal antibody that targets and binds to CLDN18.2 to mediate cancer cell death via antibody-dependent cellular cytotoxicity and complement-dependent cytotoxicity [15, 16, 18, 20, 25]. Recently, the phase 3 SPOTLIGHT (NCT03504397) and GLOW (NCT03653507) studies demonstrated statistically significant improvements in progression-free survival (PFS) and OS when zolbetuximab was combined with chemotherapy in patients with HER2-negative, LA unresectable or mG/GEJ adenocarcinoma whose tumors were CLDN18.2-positive [26, 27]. These findings established CLDN18.2 as a novel biomarker with clinical relevance in this patient population.

There are limited data on the global prevalence of CLDN18.2 positivity in tumors of patients with LA unresectable or mG/GEJ adenocarcinoma. A recent retrospective study reported the prevalence of CLDN18.2 positivity, defined as ≥ 75% of tumor cells demonstrating moderate-to-strong membranous CLDN18 staining using the on-market VENTANA CLDN18 (43-14A) Assay (VMSI/Roche), as 24.0% in a cohort of 408 evaluable patients with advanced G/GEJ adenocarcinoma from Japan [21]. Another retrospective study using the same assay and cut-off values for CLDN18.2 positivity reported a prevalence of 33.4% in a cohort of 350 evaluable White patients with advanced G/GEJ adenocarcinoma from Italy [12]. Similarly, using the same assay and cut-off values for CLDN18.2 positivity, a prevalence of 30.1% was reported in a clinical cohort of 286 evaluable patients from North America, Asia, and Europe [13].

Here we report the prevalence of CLDN18.2 positivity from more than 4000 patient tumor samples tested across the global, multicenter, phase 3 SPOTLIGHT and GLOW studies and the association of CLDN18.2 status with demographic, clinical, and histopathological characteristics. We also report the concordance of CLDN18.2 positivity in a subset of pair-matched samples consisting of archival tumor samples and baseline tumor samples from the phase 2 ILUSTRO study (NCT03505320; cohorts 1A and 2) and a phase 1 study in Japanese patients (NCT03528629) [28, 29].

## Patients and methods

### Patients

**Fig. 1:**
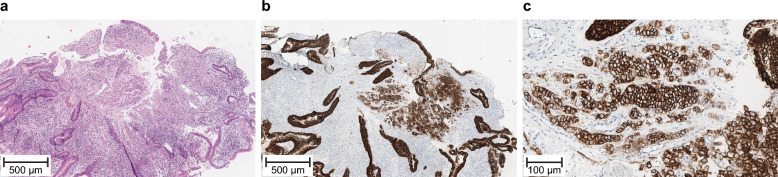
Example of CLDN18.2-positive G/GEJ tumor sample stained for CLDN18 using the VENTANA CLDN18 (43-14A) RxDx Assay (for Investigational Use Only; VMSI/Roche)^a^. **a** hematoxylin and eosin staining. **b**, **c** CLDN18.2 immunohistochemistry. ^a^CLDN18.2 positivity was defined as ≥ 75% of tumor cells demonstrating moderate-to-strong membranous CLDN18 staining. CLDN18, claudin 18; CLDN18.2, claudin 18 isoform 2; G/GEJ, gastric or gastroesophageal junction

The randomized phase 3 SPOTLIGHT and GLOW studies evaluated 1L zolbetuximab plus chemotherapy (mFOLFOX6 or CAPOX, respectively) versus placebo plus chemotherapy in patients with HER2-negative, LA unresectable or mG/GEJ adenocarcinoma whose tumors were CLDN18.2-positive, defined as ≥ 75% of tumor cells demonstrating moderate-to-strong membranous CLDN18 staining using the VENTANA CLDN18 (43-14A) RxDx Assay (for Investigational Use Only; VMSI/Roche) (Fig. 1). The study designs were published previously [26, 27, 30].

The nonrandomized phase 2 ILUSTRO study evaluated zolbetuximab with or without chemotherapy or immunotherapy in patients with LA unresectable or mG/GEJ adenocarcinoma. The study design was published previously [29]. This study analyzed patients from cohort 1A, which evaluated third-line or later zolbetuximab monotherapy in patients whose tumors were CLDN18.2-positive (defined using same criteria as SPOTLIGHT and GLOW) and cohort 2, which evaluated 1L zolbetuximab plus mFOLFOX6 in patients with HER2-negative tumors that were CLDN18.2-positive.

The nonrandomized phase 1 study in Japanese patients evaluated zolbetuximab monotherapy in patients with LA or mG/GEJ adenocarcinoma whose tumors were CLDN18.2-positive for whom no standard-of-care treatment was available or who were ineligible to receive an available standard-of-care treatment per investigator assessment. The Safety Part (Arms A and B) enrolled patients with tumors with any tumor cell demonstrating any membranous CLDN18 staining using the on-market VENTANA CLDN18 (43-14A) Assay (VMSI/Roche), and the Expansion Part enrolled patients with ≥ 75% of tumor cells demonstrating moderate-to-strong membranous CLDN18 staining. The study design was published previously [28].

### Biomarker assessments

In SPOTLIGHT and GLOW, archival (i.e., any time before treatment) tumor samples were collected by biopsy from either primary or metastatic tumor sites, excluding bone. In ILUSTRO and the phase 1 study, archival and baseline (i.e., within 3 months before first study treatment) tumor samples were collected, where possible, from either primary or metastatic tumor sites. Samples were submitted as formalin-fixed paraffin-embedded (FFPE) blocks or unstained slides. Slides were prepared by cutting serial ~ 4 μm-thick sections from FFPE tissue blocks and mounting the sections on positively charged glass slides. Before staining, the slides were air-dried completely, either at room temperature (15–25 °C) or by offline baking at 60 °C for 60 min. All samples were stored at room temperature prior to staining. All staining was performed within 6 months of sectioning.

CLDN18.2 status in SPOTLIGHT, GLOW, and ILUSTRO was tested by central immunohistochemistry (IHC) at Q^2^ Solutions (Durham, NC, USA) using the VENTANA CLDN18 (43-14A) RxDx Assay (for Investigational Use Only; VMSI/Roche) on a BenchMark ULTRA instrument (VMSI/Roche). The VENTANA CLDN18 (43-14A) RxDx Assay (for Investigational Use Only; VMSI/Roche) was intended for the qualitative detection of CLDN18 protein in FFPE human gastric adenocarcinoma tissue (including GEJ) and was analytically validated at the 75% positivity cutoff. The assay required 3 slides per case: 1 slide for CLDN18 antibody staining, 1 slide for staining with Ventana Negative Control (Monoclonal; catalog no. 760-2014/part no. 05266670001; VMSI/Roche) as a negative reagent control, and 1 slide for hematoxylin and eosin staining; in addition, each batch of case slides was stained together with a system-level control slide of human gastric tissue with intestinal metaplasia, which contained CLDN18-positive and CLDN18-negative staining elements.

CLDN18.2 status in the phase 1 study was tested by central IHC at Ventana Medical Systems, Inc. (Tucson, AZ, USA), using the on-market VENTANA CLDN18 (43-14A) Assay (VMSI/Roche).

In SPOTLIGHT, GLOW, and ILUSTRO (cohort 2), HER2 expression was tested by local evaluation; if local evaluation was not available, HER2 expression was evaluated centrally at Q^2^ Solutions according to American Society of Clinical Oncology (ASCO) guidelines.

In SPOTLIGHT and GLOW, PD-L1 expression was tested as an ad hoc analysis in samples from a subset of enrolled patients who provided informed consent and for whom samples were available for testing using the Dako PD-L1 IHC 28-8 pharmDx assay (CellCarta; Naperville, IL, USA). PD-L1 expression was measured using CPS, defined as the ratio of the number of PD-L1–positive tumor cells, lymphocytes, and macrophages to the total number of tumor cells, × 100 [21].

### Trial oversight

This study and the associated clinical studies were conducted in accordance with Good Clinical Practice, the Declaration of Helsinki, and the International Council for Harmonization of Technical Requirements for Registration of Pharmaceuticals for Human Use guidelines [26–29]. The studies conformed to the study protocols, protocol amendments, and investigator brochures [26–29]. Participants provided signed informed consent and privacy language per national regulations (e.g., Health Insurance Portability and Accountability Act [HIPAA] authorization for sites in the USA) prior to any study-related procedure and were updated if warranted by new information; informed consent and all other forms of study-related patient information were reviewed and approved by the appropriate independent ethics committee or institutional review board at each study site [26–29].

### Statistical methods

Continuous and categorical data were presented with descriptive statistics. Hypothesis testing of the relationship between CLDN18.2 status and demographic, clinical, and histopathological characteristics was performed with the chi-square test and excluded data from patients with missing, "unknown”, or “other” entries. The criteria for the concordance analysis were that patients had matching biopsy samples and met the CLDN18.2 status requirements as defined per each concordance analysis.

## Results

### Overall prevalence of CLDN18.2 positivity in SPOTLIGHT and GLOW

**Fig. 2:**
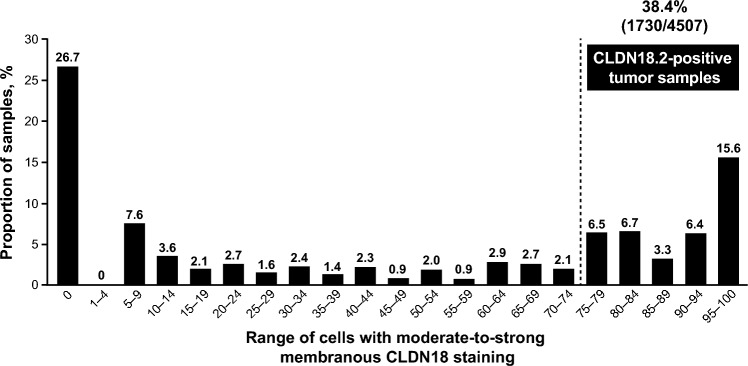
Distribution of CLDN18 staining among all screened patients in SPOTLIGHT and GLOW using the VENTANA CLDN18 (43-14A) RxDx Assay (for Investigational Use Only; VMSI/Roche). The VENTANA CLDN18 (43-14A) RxDx Assay (for Investigational Use Only; VMSI/Roche) was used in an exploratory analysis to investigate CLDN18 staining for the combined screened patient populations with LA unresectable or mG/GEJ adenocarcinoma from the SPOTLIGHT and GLOW studies. Numbers above each bar indicate the proportion of samples that fall in each bin. The dotted line indicates the range cut-off for CLDN18.2 positivity (≥ 75% of tumor cells demonstrating moderate-to-strong membranous CLDN18 staining). Bins represent range cut-offs of the percentage of cells demonstrating moderate-to-strong membranous CLDN18 staining. The sum of the proportion of samples in the 5 bins ranging from 75 to 100 was 38.5% due to rounding within individual bins. CLDN18, claudin 18; CLDN18.2, claudin 18 isoform 2; LA, locally advanced; mG/GEJ, metastatic gastric or gastroesophageal junction

An overview of the assessment and random assignment of patients in the SPOTLIGHT and GLOW studies is presented in Supplementary Fig. S1a–b, and representativeness of all screened patients with valid CLDN18 IHC results relative to the overall population of patients with advanced G/GEJ adenocarcinoma is provided in Supplementary Table S1. Across SPOTLIGHT and GLOW, the combined prevalence of CLDN18.2 positivity, defined as ≥ 75% of tumor cells demonstrating moderate-to-strong membranous CLDN18 staining using the VENTANA CLDN18 (43-14A) RxDx Assay (for Investigational Use Only; VMSI/Roche), was 38.4% (1730/4507) among all screened patients with valid CLDN18 IHC results (Table 1 and Fig. 2). The prevalence of CLDN18.2 positivity was 38.4% (922/2403) in SPOTLIGHT and 38.4% (808/2104) in GLOW.

**Table 1:** Prevalence of CLDN18.2 positivity by demographic characteristics among all screened patients in SPOTLIGHT and GLOW

Patients, n (%)	Combined			SPOTLIGHT			GLOW		
	CLDN18.2-positive	CLDN18.2-negative	*P* value	CLDN18.2-positive	CLDN18.2-negative	*P* value	CLDN18.2-positive	CLDN18.2-negative	*P* value
Overall	1730 (38.4)	2777 (61.6)		922 (38.4)	1481 (61.6)		808 (38.4)	1296 (61.6)	
Sex			< 0.001			< 0.001			0.008
Male	1093 (36.2)	1924 (63.8)		588 (36.1)	1041 (63.9)		505 (36.4)	883 (63.6)	
Female	637 (42.8)	853 (57.2)		334 (43.2)	440 (56.8)		303 (42.3)	413 (57.7)	
Race			< 0.001			< 0.001			0.101
White	772 (42.3)	1055 (57.7)		477 (43.1)	631 (56.9)		295 (41.0)	424 (59.0)	
Black or African American	15 (30.0)	35 (70.0)		13 (27.1)	35 (72.9)		2 (100.0)	0	
Asian	783 (36.4)	1366 (63.6)		288 (35.3)	528 (64.7)		495 (37.1)	838 (62.9)	
American Indian or Alaska Native	37 (31.1)	82 (68.9)		36 (31.3)	79 (68.7)		1 (25.0)	3 (75.0)	
Native Hawaiian or Other Pacific Islander	0	3 (100.0)		0	1 (100.0)		0	2 (100.0)	
Other	36 (31.0)	80 (69.0)		36 (33.0)	73 (67.0)		0	7 (100.0)	
Age group 1			< 0.001			0.005			< 0.001
≤ 65 years	1136 (40.9)	1639 (59.1)		572 (40.7)	832 (59.3)		564 (41.1)	807 (58.9)	
> 65 years	594 (34.3)	1138 (65.7)		350 (35.0)	649 (65.0)		244 (33.3)	489 (66.7)	
Age group 2			0.002			0.018			0.06
≤ 75 years	1619 (39.0)	2529 (61.0)		849 (39.1)	1320 (60.9)		770 (38.9)	1209 (61.1)	
> 75 years	111 (30.9)	248 (69.1)		73 (31.2)	161 (68.8)		38 (30.4)	87 (69.6)	

CLDN18.2, claudin 18 isoform 2

The prevalence of CLDN18.2 positivity trended slightly higher in screened patients with HER2-negative tumors compared with all screened patients as described above. Among screened patients with HER2-negative tumors based on central testing with valid CLDN18 IHC results across SPOTLIGHT and GLOW, the combined prevalence of CLDN18.2 positivity was 42.3% (1568/3705). The prevalence of CLDN18.2 positivity in patients with HER2-negative tumors was 41.9% (839/2004) in SPOTLIGHT and 42.9% (729/1701) in GLOW.

Among screened patients with HER2-positive tumors based on central testing and valid CLDN18 IHC results across SPOTLIGHT and GLOW, the combined prevalence of CLDN18.2 positivity was 28.8% (124/430). The prevalence of CLDN18.2 positivity in patients with HER2-positive tumors was 26.2% (60/229) in SPOTLIGHT and 31.8% (64/201) in GLOW.

### Prevalence of CLDN18.2 positivity by demographic characteristics in SPOTLIGHT and GLOW

Across SPOTLIGHT and GLOW, a statistically significant association was observed between CLDN18.2 status and sex (*P* < 0.001): the prevalence of CLDN18.2 positivity was higher in tumors from female patients (42.8%; 637/1490) compared with tumors from male patients (36.2%; 1093/3017) (Table 1). A statistically significant association was also observed between CLDN18.2 status and race (*P* < 0.001): the prevalence of CLDN18.2 positivity was highest in tumors from White patients (42.3%; 772/1827) and from Asian patients (36.4%; 783/2149) (Table 1). A statistically significant association was observed between CLDN18.2 status and age at 2 cut-offs (age group 1, ≤ 65 vs > 65 years of age, *P* < 0.001; age group 2, ≤ 75 vs > 75 years of age, *P* = 0.002); the prevalence of CLDN18.2 positivity was higher in tumors from patients ≤ 65 years of age (40.9%; 1136/2775) compared with > 65 years of age (34.3%; 594/1732), and from patients ≤ 75 years of age (39.0%; 1619/4148) compared with > 75 years of age (30.9%; 111/359) (Table 1).

### Prevalence of CLDN18.2 positivity by geographical region in SPOTLIGHT and GLOW

**Fig. 3:**
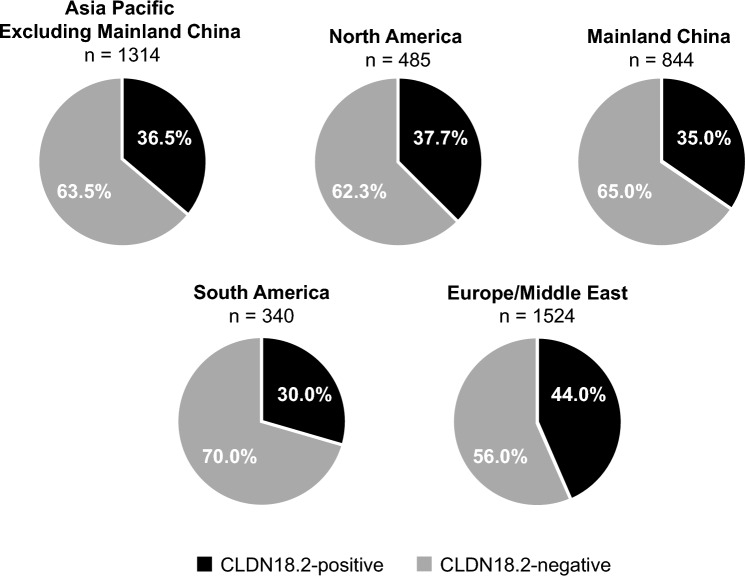
Prevalence of CLDN18.2 positivity^a^ among all screened patients in SPOTLIGHT and GLOW by region. ^a^CLDN18.2 positivity was defined as ≥ 75% of tumor cells demonstrating moderate-to-strong membranous CLDN18 staining using the VENTANA CLDN18 (43-14A) RxDx Assay (for Investigational Use Only; VMSI/Roche). CLDN18, claudin 18; CLDN18.2, claudin 18 isoform 2

Across SPOTLIGHT and GLOW, the prevalence of CLDN18.2 positivity ranged from approximately 30%–44% across geographic regions (Fig. 3); regions are outlined in Supplementary Table S2. The prevalence of CLDN18.2 positivity was 36.5% (479/1314) in tumors from patients in the Asia–Pacific region, excluding mainland China; 37.7% (183/485) in tumors from patients in North America; 35.0% (295/844) in tumors from patients in mainland China; 30.0% (102/340) in tumors from patients in South America; and 44.0% (671/1524) in tumors from patients in Europe and the Middle East (Fig. 3).

Similar to the observation in the overall population, the prevalence of CLDN18.2 positivity in each geographic region trended slightly higher in patients with HER2-negative tumors compared with the prevalence observed in each geographic region in tumors from all screened patients (Supplementary Fig. S2). The prevalence of CLDN18.2 positivity in patients with HER2-positive tumors was not assessed by geographic region.

### Prevalence of CLDN18.2 positivity by clinical and histopathological characteristics in SPOTLIGHT and GLOW

There was no association observed between CLDN18.2 status and medical condition across SPOTLIGHT and GLOW (*P* = 0.18): the prevalence of CLDN18.2 positivity was 39.9% (1341/3357) in tumors from patients with gastric adenocarcinoma and 37.5% (338/901) in tumors from patients with GEJ adenocarcinoma (Table 2). A statistically significant association was observed between CLDN18.2 status and Lauren classification (*P* < 0.001): the prevalence of CLDN18.2 positivity was higher in diffuse-type tumors (48.3%; 553/1145) compared with intestinal-type tumors (38.8%; 308/794) or mixed-type tumors (42.9%; 134/312) (Table 2).

**Table 2:** Prevalence of CLDN18.2 positivity by disease characteristics among all screened patients in SPOTLIGHT and GLOW

Patients, n (%)	Combined			SPOTLIGHT			GLOW		
	CLDN18.2-positive	CLDN18.2-negative	*P* value	CLDN18.2-positive	CLDN18.2-negative	*P* value	CLDN18.2-positive	CLDN18.2-negative	*P* value
Overall	1730 (38.4)	2777 (61.6)		922 (38.4)	1481 (61.6)		808 (38.4)	1296 (61.6)	
Medical condition			0.18			0.14			0.81
Gastric	1341 (39.9)	2016 (60.1)		673 (40.1)	1004 (59.9)		668 (39.8)	1012 (60.2)	
GEJ	338 (37.5)	563 (62.5)		217 (36.7)	374 (63.3)		121 (39.0)	189 (61.0)	
Gastric tumor location			0.56			0.49			0.90
Proximal	393 (44.0)	501 (56.0)		187 (43.9)	239 (56.1)		206 (44.0)	262 (56.0)	
Distal	502 (45.3)	607 (54.7)		255 (46.1)	298 (53.9)		247 (44.4)	309 (55.6)	
Unknown	438 (33.5)	869 (66.5)		223 (34.3)	428 (65.7)		215 (32.8)	441 (67.2)	
Lauren classification			< 0.001			0.10			< 0.001
Diffuse	553 (48.3)	592 (51.7)		301 (48.5)	319 (51.5)		252 (48.0)	273 (52.0)	
Intestinal	308 (38.8)	486 (61.2)		186 (42.4)	253 (57.6)		122 (34.4)	233 (65.6)	
Mixed	134 (42.9)	178 (57.1)		68 (42.8)	91 (57.2)		66 (43.1)	87 (56.9)	
Other	252 (37.2)	425 (62.8)		150 (36.6)	260 (63.4)		102 (38.2)	165 (61.8)	
Unknown	412 (35.0)	765 (65.0)		166 (34.0)	322 (66.0)		246 (35.7)	443 (64.3)	

CLDN18.2, claudin 18 isoform 2; GEJ, gastroesophageal junction

There was no association observed between CLDN18.2 status and the method of tumor collection across SPOTLIGHT and GLOW (*P* = 0.57): the prevalence of CLDN18.2 positivity was 38.6% (1403/3632) in tumor samples collected by biopsy and 37.6% (327/870) in tumor samples collected by resection (Table 3). There was no association observed between CLDN18.2 status and the site of tumor collection (*P* = 0.55): the prevalence of CLDN18.2 positivity was 38.0% (1498/3939) in samples collected from primary sites and 39.4% (201/510) in samples collected from metastatic sites (Table 3).

**Table 3:** Prevalence of CLDN18.2 positivity by tumor sample characteristics among all screened patients in SPOTLIGHT and GLOW

Patients, n (%)	Combined			SPOTLIGHT			GLOW		
	CLDN18.2-positive	CLDN18.2-negative	*P* value	CLDN18.2-positive	CLDN18.2-negative	*P* value	CLDN18.2-positive	CLDN18.2-negative	*P* value
Overall	1730 (38.4)	2777 (61.6)		922 (38.4)	1481 (61.6)		808 (38.4)	1296 (61.6)	
Collection method			0.57			0.47			0.11
Biopsy	1403 (38.6)	2229 (61.4)		741 (38.0)	1207 (62.0)		662 (39.3)	1022 (60.7)	
Resection	327 (37.6)	543 (62.4)		181 (39.9)	273 (60.1)		146 (35.1)	270 (64.9)	
Unknown	0	5 (100.0)		0	1 (100.0)		0	4 (100.0)	
Collection site			0.55			0.16			0.46
Primary	1498 (38.0)	2441 (62.0)		762 (37.4)	1273 (62.6)		736 (38.7)	1168 (61.3)	
Metastatic	201 (39.4)	309 (60.6)		129 (41.6)	181 (58.4)		72 (36.0)	128 (64.0)	

CLDN18.2, claudin 18 isoform 2

### Prevalence of PD-L1 CPS ≥ 5 in SPOTLIGHT and GLOW

As an ad hoc analysis, PD-L1 expression was evaluated in a subset of enrolled patients (whose tumors were CLDN18.2-positive) in SPOTLIGHT and GLOW. Across SPOTLIGHT and GLOW, a PD-L1 CPS ≥ 5 was observed in the tumors of 17.4% (104/599) of patients (Supplementary Table S3). A PD-L1 CPS ≥ 5 was observed in the tumors of 13.2% (41/311) of patients in SPOTLIGHT and 21.9% (63/288) of patients in GLOW. Across SPOTLIGHT and GLOW, a PD-L1 CPS < 5 was observed in the tumors of 82.6% (495/599) of patients. A PD-L1 CPS < 5 was observed in the tumors of 86.8% (270/311) of patients in SPOTLIGHT and 78.1% (225/288) of patients in GLOW.

### Concordance of CLDN18.2 positivity in the phase 2 ILUSTRO study and the phase 1 study

An overview of the assessment and allocation of patients in the ILUSTRO study and the phase 1 study is presented in Supplementary Fig. S1c–d. Across cohorts 1A and 2 of ILUSTRO and the phase 1 study, the prevalence of CLDN18.2 positivity was 36.1% (150/416) [31]. As an exploratory analysis, the concordance of CLDN18.2 positivity (defined as ≥ 75% of tumor cells demonstrating moderate-to-strong membranous CLDN18 staining) or the concordance of any CLDN18 staining (defined as ≥ 1% of tumor cells demonstrating moderate-to-strong membranous CLDN18 staining) was assessed in a subset of pair-matched samples consisting of archival tumor samples, which were collected any time before treatment, and baseline tumor samples, which were collected within 3 months before first study treatment (cohorts 1A and 2 of the ILUSTRO study, *n* = 23; phase 1 study, *n* = 13). The median time between collection of archival tumor samples and baseline tumor samples was 415 days (range, 19–2732). Across ILUSTRO and the phase 1 study, the concordance rate (overall percentage agreement) of CLDN18.2 positivity between archival and baseline tumor samples was 61.1% (22/36) (Supplementary Table S4). The concordance rate of any CLDN18 staining between archival and baseline tumor samples was 88.9% (32/36) (Supplementary Table S5). Individual patient data are presented in Supplementary Table S6.

## Discussion

This study evaluated the prevalence of CLDN18.2 positivity, defined as ≥ 75% of tumor cells demonstrating moderate-to-strong membranous CLDN18 staining using the VENTANA CLDN18 (43-14A) RxDx Assay (for Investigational Use Only; VMSI/Roche), and the association of CLDN18.2 status with demographic, clinical, and histopathological characteristics in tumors from patients with HER2-negative, LA unresectable or mG/GEJ adenocarcinoma. The prevalence of CLDN18.2 positivity was consistent across SPOTLIGHT (38.4%) and GLOW (38.4%). A statistically significant association was observed between CLDN18.2 status and the sex, race, and age of patients from whom tested tumors were collected. A statistically significant association was also observed between CLDN18.2 status and the Lauren classification of tested tumors; although CLDN18.2 positivity was most prevalent in patients with diffuse-type tumors (48.3%), the prevalence of CLDN18.2 positivity was also high in patients with intestinal-type (38.8%) and mixed-type tumors (42.9%), suggesting that tumors from patients with G/GEJ adenocarcinoma should be tested for CLDN18.2 positivity regardless of disease histology. While some previous studies of patients with various stages of G/GEJ adenocarcinoma have shown an association between CLDN18.2 status and a diffuse-type Lauren classification [17, 32, 33], recent studies using the same diagnostic antibody and cut-off for CLDN18.2 positivity as used for the current study demonstrated similar prevalence of CLDN18.2 positivity in intestinal-type and diffuse-type tumors [12, 13, 21]. Further research is needed to establish if there is a link between CLDN18.2 status and Lauren classification in patients with LA unresectable or mG/GEJ adenocarcinoma. It is important to note that, while statistically significant associations of CLDN18.2 status with multiple patient and tumor characteristics were observed, the numerical differences in the prevalence of CLDN18.2 positivity between groups were generally small, suggesting that G/GEJ tumors broadly have membranous CLDN18.2.

No associations were observed between CLDN18.2 status and the medical condition (i.e., gastric versus GEJ adenocarcinoma) of patients from whom tested tumors were collected, the method by which tumors were collected, or the site from which tumors were collected. In a retrospective study of a cohort of Italian patients using the same diagnostic antibody and criteria for CLDN18.2 positivity, a concordance rate of 81.5% was reported in pair-matched tumor samples from 27 patients collected from primary and metastatic sites [12]. Though further research is needed, our study suggests that tumor samples collected by biopsy or resection and from primary or metastatic sites can be reliably used to screen for CLDN18.2 positivity, defined as ≥ 75% of tumor cells demonstrating moderate-to-strong membranous CLDN18 staining using the VENTANA CLDN18 (43-14A) RxDx Assay (for Investigational Use Only; VMSI/Roche), in patients with LA unresectable or mG/GEJ adenocarcinoma.

This study evaluated the prevalence of tumors with a PD-L1 CPS ≥ 5 in a subset of randomly assigned patients (i.e., whose tumors were CLDN18.2-positive) in SPOTLIGHT and GLOW. Across SPOTLIGHT and GLOW, 17.4% of assessed patients had tumors with a PD-L1 CPS ≥ 5. In previous studies, the prevalence of tumors with a PD-L1 CPS ≥ 5 independent of CLDN18.2 status ranged from approximately 20%–60% [9, 12, 21]. In a recent clinical study of first-line pembrolizumab plus chemotherapy in patients with HER2-negative, LA unresectable or mG/GEJ adenocarcinoma (KEYNOTE-859, NCT03675737), 34.9% of enrolled patients had tumors with a PD-L1 CPS ≥ 10 [34]. In a recent clinical study of nivolumab plus chemotherapy in patients with advanced unresectable or metastatic gastric, GEJ, or esophageal adenocarcinoma with no known HER2-positivity (CheckMate 649, NCT02872116), 60% of randomly assigned patients had tumors with a PD-L1 CPS ≥ 5 [9]. It is possible that there are fewer patients with tumors with a high PD-L1 CPS among patients whose tumors are CLDN18.2-positive compared with patients whose tumors are CLDN18.2-negative, resulting in the observed difference in the prevalence of tumors with a high PD-L1 CPS in SPOTLIGHT and GLOW versus KEYNOTE-859 and CheckMate 649. However, 2 recent retrospective studies using the same diagnostic antibody and cut-off for CLDN18.2 positivity suggested that the prevalence of tumors with a PD-L1 CPS ≥ 5 is similar in patients whose tumors are CLDN18.2-positive and whose tumors are CLDN18.2-negative (17.9% vs 21.5% with a PD-L1 CPS ≥ 5, respectively, in a cohort of Italian patients; 41.9% vs 51.5% with a PD-L1 CPS ≥ 5 in a cohort of Japanese patients) [12, 21]. It is unclear if the prevalence of tumors with a PD-L1 CPS ≥ 5, which was lower in SPOTLIGHT and GLOW compared with other studies, is related to factors such as local prescreening of patients for PD-L1 expression, which warrants further study. These studies also did not observe a difference in HER2-expression status or mismatch-repair status in patients whose tumors were CLDN18.2-positive compared to patients whose tumors were CLDN18.2-negative [12, 21]. Together, these studies suggest that CLDN18.2 is a prevalent biomarker that defines a new subgroup of patients whose tumors are CLDN18.2-positive who may benefit from treatment with zolbetuximab.

This study also evaluated the concordance of CLDN18.2 positivity (defined as ≥ 75% of tumor cells demonstrating moderate-to-strong membranous CLDN18 staining) or any CLDN18 staining (defined as ≥ 1% of tumor cells demonstrating moderate-to-strong membranous CLDN18 staining) in a subset of pair-matched samples consisting of archival tumor samples (collected any time before treatment) and baseline tumor samples (collected within 3 months before first study treatment) from the ILUSTRO study (cohorts 1A and 2) and the phase 1 study of Japanese patients. The median time between collection of samples was over 1 year (415 days), during which patients may have received many different treatments. The concordance rate of CLDN18.2 positivity was 61.1%, and the concordance rate of any CLDN18 staining was 88.9%. In a retrospective study in a cohort of Japanese patients using the same diagnostic antibody and criteria for CLDN18.2 positivity, a concordance rate of 75.1% was reported between pair-matched tumor samples from 17 patients collected before and after 1L chemotherapy [21]. In another retrospective study in a cohort of Italian patients using the same diagnostic antibody and criteria for CLDN18.2 positivity, a concordance rate of 66.7% was reported between pair-matched primary tumor samples from 27 patients collected by biopsy and by surgery [12]. In the current study, the nonconcordance of CLDN18.2 positivity between archival and baseline tumor samples in 38.9% of patients may possibly have been due to (1) effects of previous therapies, (2) intertumoral heterogeneity between samples collected from primary sites and samples collected from metastatic sites, or (3) as noted previously, intratumoral heterogeneity between archival and baseline samples collected from the same tumor [21]. This is the first analysis of the concordance of CLDN18.2 positivity in archival versus baseline tumor samples; although concordance was high in this study, future research may further clarify the reasons for nonconcordance observed in some patient tumors. Together, these studies suggest that CLDN18.2 is an antigen that remains relatively stable over long periods of time in many patients.

A strength of this analysis is that the SPOTLIGHT and GLOW studies represent the largest sources to date for data on the global prevalence of CLDN18.2 positivity in patients with HER2-negative, LA unresectable or mG/GEJ adenocarcinoma. SPOTLIGHT and GLOW enrolled a diverse, global patient population, spanning 5 geographic regions and 30 countries.

A limitation of this study is that despite the large patient population, these observations may not be generalizable to all patients with HER2-negative, LA unresectable or mG/GEJ adenocarcinoma. Additional research on the prevalence of CLDN18.2 positivity in patients following biomarker testing in the real-world setting is needed to refine the understanding of the prevalence of this biomarker in the global patient population. Additionally, the number of samples analyzed for concordance of CLDN18.2 positivity was relatively small, and additional research is needed to understand the stability of CLDN18.2 status in tumors over time. This study did not collect information on the presence of signet-ring cells or evaluate a potential association between this disease subtype and prevalence of CLDN18.2 positivity in patient tumor samples; however, previous studies have suggested a high prevalence of CLDN18.2 positivity and *CLDN18* expression in signet-ring cell carcinoma [35, 36]. This study also did not evaluate the prevalence of CLDN18.2 positivity in Epstein-Barr virus (EBV)-positive or EBV-negative tumors. Some previous studies have suggested that the prevalence of CLDN18.2 positivity is higher in patients with EBV-positive versus EBV-negative tumors; however, one recent study suggested that the prevalence of CLDN18.2 positivity is not enriched in patients with EBV-positive tumors [21, 33, 37, 38].

This study demonstrated that CLDN18.2 is a highly prevalent and relatively stable biomarker. The results suggest that CLDN18 can be reliably detected in patient tumor samples regardless of the collection method or site using the VENTANA CLDN18 (43-14A) RxDx Assay (for Investigational Use Only; VMSI/Roche). The results also suggest that CLDN18.2 positivity is highly prevalent in all assessed histologic subtypes of G/GEJ adenocarcinoma; like any other biomarker tested by IHC, testing more samples per patient will give more accurate results as to account for tumor heterogeneity. This study supports that testing for CLDN18.2 positivity can aid in identifying patients who may benefit from CLDN18.2-targeted therapy with zolbetuximab, and that testing for CLDN18.2 positivity in all histologic subtypes should be incorporated into routine biomarker testing along with testing for HER2, PD-L1, and microsatellite instability for patients with LA unresectable or mG/GEJ adenocarcinoma.

### Supplementary Information

Below is the link to the electronic supplementary material.

Supplementary file1 (PDF 441 KB)

## Data Availability

Upon request, and subject to certain criteria, conditions, and exceptions, Astellas will provide access to anonymized patient level data from completed Astellas sponsored phase 1–4 interventional clinical studies conducted for its products. Where available, the following anonymized patient level data and information is provided for each clinical study: raw dataset, analysis ready dataset, protocols with any amendments or addenda, annotated case report form, statistical analysis plan, dataset specifications, and clinical study report. Additional data may be available upon request. Researchers may request access to anonymized participant level data, trial level data, and protocols from Astellas sponsored clinical trials by submitting a query at: www.clinicalstudydatarequest.com. For the Astellas criteria on data sharing see: https://clinicalstudydatarequest.com/Study-Sponsors/Study-Sponsors-Astellas.aspx.
