# Supplementary material for: Global prevalence of claudin 18 isoform 2 in tumors of patients with locally advanced unresectable or metastatic gastric or gastroesophageal junction adenocarcinoma
Source: Gastric Cancer. 2024 Jul 2;27(5):1058–68. doi: 10.1007/s10120-024-01518-1 (PMC11335819; doi:10.1007/s10120-024-01518-1)
Supplement: Supplementary file 1 — Supplementary file1 (PDF 441 KB) [file 10120_2024_1518_MOESM1_ESM.pdf]

Global prevalence of claudin 18 isoform 2 in tumors of patients with locally advanced unresectable or metastatic gastric or gastroesophageal junction adenocarcinoma

Kohei Shitara[1]*, Rui-Hua Xu[2]*, Jaffer A. Ajani[3], Diarmuid Moran[4], Abraham Guerrero[4], Ran Li[4], Janet Pavese[4], Maria Matsangou[4], Pranob Bhattacharya[4], Yoko Ueno[5], Xuewei Wang[4], Manish A. Shah[6]

*These authors are co-lead authors.

[1]Department of Gastrointestinal Oncology, National Cancer Center Hospital East, Kashiwa City, Chiba, Japan; [2]Department of Medical Oncology, Sun Yat-Sen University Cancer Center, State Key Laboratory of Oncology in South China, Collaborative Innovation Center for Cancer Medicine, Guangzhou, China; [3]Department of Gastrointestinal Medical Oncology, Division of Cancer Medicine, The University of Texas, MD Anderson Cancer Center, Houston, TX, USA; [4]Astellas Pharma Global Development, Inc., Northbrook, IL, USA; [5]Astellas Pharma Inc., Tokyo, Japan; [6]Division of Hematology and Medical Oncology, Weill Cornell Medical College, New York City, NY, USA

Corresponding author

Manish A. Shah, MD

Telephone number: 646-962-6200

Email: mas9313@med.cornell.edu

ORCID: 0000-0002-6913-9655

Table of Contents

Table S1. Representativeness of study participants. 3

Table S2. Representative geographic regions and associated countries in SPOTLIGHT and GLOW. 7

Table S3. PD-L1 analysis in a subset of enrolled patients (whose tumors were CLDN18.2-positive) in SPOTLIGHT and GLOW. 8

Table S4. Concordance of CLDN18.2 positivity[a] between archival and baseline tumor samples in ILUSTRO (cohorts 1A and 2) and the phase 1 study. 9

Table S5. Concordance of any CLDN18 staining[a] between archival and baseline tumor samples in ILUSTRO (cohorts 1A and 2) and the phase 1 study. 10

Table S6. CLDN18 staining in individual patients in ILUSTRO and the phase 1 study. 11

Figure S1. CONSORT diagrams of patient populations in SPOTLIGHT, GLOW, ILUSTRO, and the phase 1 study. 13

Figure S2. Prevalence of CLDN18.2 positivity[a] among all screened patients with HER2-negative tumors in SPOTLIGHT and GLOW by region. 15

Supplementary references 16

Table S1. Representativeness of study participants.

**Cancer type(s)/subtype(s)/ stage(s)/condition**	Patients with previously untreated, HER2-negative, LA unresectable or mG/GEJ adenocarcinoma whose tumors were CLDN18.2-positive
**Considerations related to:**	
**Sex**	Of patients with valid CLDN18 IHC results across the global SPOTLIGHT and GLOW studies, 66.9% (3017/4507) were male.<br><br>In 2 recent retrospective studies of CLDN18.2 status in Japanese and Italian patients with advanced G/GEJ adenocarcinoma, 68.9% (281/408) and 62.3% (218/350) patients were male, respectively [1, 2].<br><br>In the global CheckMate 649 study of patients with previously untreated, advanced unresectable or metastatic gastric, GEJ, or esophageal adenocarcinoma with no known HER2-positivity, 69.6% (1100/1581) of patients were male [3].<br><br>The Global Cancer Statistics (2020) identifies gastric cancer as twice as common in men compared with women [4].
**Age**	Of patients with valid CLDN18 IHC results across the global SPOTLIGHT and GLOW studies, 61.6% (2775/4507) were ≤65 years of age.<br><br>In 2 recent retrospective studies of CLDN18.2 status in Japanese and Italian patients with advanced G/GEJ adenocarcinoma, 41.4% (169/408) of patients were <65 years of age, and 59.7% (209/350) were <70 years of age, respectively [1, 2].

	In the global CheckMate 649 study of patients with previously untreated, advanced unresectable or metastatic gastric, GEJ, or esophageal adenocarcinoma with no known HER2-positivity, 60.8% (961/1581) of patients were <65 years of age [3].
**Race[a]**	Of patients with valid CLDN18 IHC results across the global SPOTLIGHT and GLOW studies for whom Race was reported, 50.4% (2149/4264) were Asian, 42.8% (1827/4264) were White, 2.8% (119/4264) were American Indian or Alaska Native, 1.2% (50/4264) were Black or African American, 0.1% (3/4264) were Native Hawaiian or Other Pacific Islander, and 2.7% (116/4264) identified as an Other Race category.<br><br>In the global CheckMate 649 study of patients with previously untreated, advanced unresectable or metastatic gastric, GEJ, or esophageal adenocarcinoma with no known HER2-positivity, for whom Race was reported, 69.4% (1097/1580) were White, 23.7% (375/1580) were Asian, 1.6% (26/1580) were American Indian or Alaska Native, 1.1% (18/1580) were Black or African American, and 4.1% (64/1580) identified as an Other Race category [3].
**Geography**	Of patients with valid CLDN18 IHC results across the global SPOTLIGHT and GLOW studies, 29.2% (1314/4507) were from Asia Pacific excluding Mainland China, 18.7% (844/4507) were from Mainland China, 33.8% (1524/4507) were from Europe or the Middle East, 10.8% (485/4507) were from North America, and 7.5% (340/4507) were from South America.<br><br>The Global Cancer Statistics (2020) estimates of cancer incidence and mortality produced by the International Agency for Research on Cancer identifies the highest incidence rates

	of gastric cancer in Eastern Asia and Eastern Europe, with lower rates in North America, Northern Europe, and Africa [4].
**Medical condition**[b]	Of patients with valid CLDN18 IHC results across the global SPOTLIGHT and GLOW studies for whom the primary site of disease was reported, 78.8% (3357/4258) had gastric adenocarcinoma.<br><br>In 2 recent retrospective studies of CLDN18.2 expression in Japanese and Italian patients with advanced G/GEJ adenocarcinoma, 89.0% (363/408) and 80.0% (280/350) patients had gastric adenocarcinoma, respectively [1, 2].<br><br>In the global CheckMate 649 study of patients with previously untreated, advanced unresectable or metastatic gastric, GEJ, or esophageal adenocarcinoma with no known HER2-positivity, among patients with gastric or GEJ tumors, 81.0% (1110/1370) had gastric adenocarcinoma[c] [3].
**PD-L1 expression**	Across SPOTLIGHT and GLOW, 17.4% of assessed patients had tumors with a PD-L1 CPS ≥5.<br><br>In previous studies, the prevalence of tumors with a PD-L1 CPS ≥5 independent of CLDN18.2 status ranged from approximately 20%–60% [1-3].<br><br>In the recent KEYNOTE-859 study of patients with LA unresectable or mG/GEJ adenocarcinoma, 34.9% of enrolled patients had tumors with a PD-L1 CPS ≥10 [5].

	In the global CheckMate 649 study of patients with previously untreated, advanced unresectable or metastatic gastric, GEJ, or esophageal adenocarcinoma with no known HER2-positivity, 60% of randomly assigned patients had tumors with a PD-L1 CPS ≥5 [3].
**Overall representativeness of this study**	In the SPOTLIGHT and GLOW studies, among all screened patients with valid CLDN18 IHC results, the patient sex, age, race, geographic origin, and primary site of disease was consistent with other recent regional retrospective and global clinical studies. In a subset of patients with CLDN18.2-positive tumors enrolled in these studies, the prevalence of G/GEJ tumors with a PD-L1 CPS ≥5 was lower than in previously published studies, and possible reasons are enumerated in the Discussion. These two phase 3 studies are the largest data source to date for characterizing the prevalence of CLDN18.2 positivity, with SPOTLIGHT enrolling patients from 215 sites across 20 countries and GLOW enrolling patients from 166 sites across 18 countries [6, 7].

[a]Race was not reported for 206 patients in SPOTLIGHT and 37 patients in GLOW.

[b]The medical condition (gastric versus GEJ adenocarcinoma) was not reported for 135 patients in SPOTLIGHT and 114 patients in GLOW.

[c]Patients with esophageal adenocarcinoma from CheckMate 649 were excluded here for purpose of comparison.

CLDN18, claudin 18; CLDN18.2, claudin 18 isoform 2; CPS, combined positive score; G/GEJ, gastric or gastroesophageal junction; HER2, human epidermal growth factor receptor 2; IHC, immunohistochemistry; LA, locally advanced; mG/GEJ, metastatic gastric or gastroesophageal junction; PD-L1, programmed cell death ligand 1.

Table S2. Representative geographic regions and associated countries in SPOTLIGHT and GLOW.

Region	Countries	
	**SPOTLIGHT**	**GLOW**
Asia Pacific excluding mainland China	• Australia<br>• Japan<br>• Republic of Korea	• Japan<br>• Malaysia<br>• Republic of Korea<br>• Thailand
North America	• Canada<br>• Mexico<br>• United States	• Canada<br>• United States
Mainland China	• Mainland China<br>• Taiwan	• Mainland China<br>• Taiwan
South America	• Brazil<br>• Chile<br>• Columbia<br>• Peru	• Argentina
Europe and Middle East	• Belgium<br>• France<br>• Germany<br>• Israel<br>• Italy<br>• Poland<br>• Spain<br>• United Kingdom	• Croatia<br>• Greece<br>• Ireland<br>• Netherlands<br>• Portugal<br>• Romania<br>• Spain<br>• Turkey<br>• United Kingdom

Table S3. PD-L1 analysis in a subset of enrolled patients (whose tumors were CLDN18.2-positive) in SPOTLIGHT and GLOW.

PD-L1 CPS, n (%)	Combined	SPOTLIGHT	GLOW
≥5	104 (17.4)	41 (13.2)	63 (21.9)
<5	495 (82.6)	270 (86.8)	225 (78.1)

CLDN18.2, claudin 18 isoform 2; CPS, combined positive score; PD-L1, programmed cell death ligand 1.

Table S4. Concordance of CLDN18.2 positivity[a] between archival and baseline tumor samples in ILUSTRO (cohorts 1A and 2) and the phase 1 study.

Archival samples[b]	Baseline samples[c]		Total (n)
	**CLDN18.2-positive[a] (n)**	**CLDN18.2-negative (n)**	
**CLDN18.2-positive[a] (n)**	20	14	34
**CLDN18.2-negative (n)**	0	2	2
**Total (n)**	20	16	36
**Concordance rate[d] (%)**	61.1		

[a]CLDN18.2 positivity was defined as ≥75% of tumor cells demonstrating moderate-to-strong membranous CLDN18 staining.

[b]Archival samples were collected any time before treatment.

[c]Baseline samples were collected within 3 months before first study treatment.

[d]Overall percentage agreement.

CLDN18, claudin 18; CLDN18.2, claudin 18 isoform 2.

Table S5. Concordance of any CLDN18 staining[a] between archival and baseline tumor samples in ILUSTRO (cohorts 1A and 2) and the phase 1 study.

Archival samples[b]	Baseline samples[c]		Total (n)
	Any CLDN18 staining[a] (n)	No CLDN18 staining (n)	
**Any CLDN18 staining[a] (n)**	32	1	33
**No CLDN18 staining (n)**	3	0	3
**Total (n)**	35	1	36
**Concordance rate[d](%)**	88.9		

[a]Any CLDN18 staining was defined as ≥1% of tumor cells demonstrating moderate-to-strong membranous CLDN18 staining.

[b]Archival samples were collected any time before treatment.

[c]Baseline samples were collected within 3 months before first study treatment.

[d]Overall percentage agreement.

CLDN18, claudin 18; CLDN18.2, claudin 18 isoform 2.

Table S6. CLDN18 staining in individual patients in ILUSTRO and the phase 1 study.

	Tumor cells demonstrating moderate-to-strong membranous CLND18 staining (%)		
**Patient**	**Archival samples[a]**	**Baseline samples[b]**	**Time between archival and baseline sample collection (days)**
1	100	100	270
2	90	30	70
3	85	90	267
4	95	89	341
5	90	80	2732
6	95	0	62
7	100	70	1255
8	100	95	573
9	90	45	195
10	95	3	945
11	80	70	1306
12	100	100	653
13	75	90	1193
14	80	80	534
15	80	0	509
16	80	100	728
17	95	100	1133
18	75	35	803
19	75	85	459
20	80	100	830
21	85	80	666
22	75	95	157

23	80	65	351
24	75	90	374
25	0	30	581
26	95	85	140
27	10	60	27
28	100	95	21
29	94	80	546
30	90	70	187
31	75	10	19
32	99	85	84
33	89	3	175
34	90	5	264
35	99	95	455
36	75	0	76

[a]Archival samples were collected any time before treatment.

[b]Baseline samples were collected within 3 months before first study treatment.

CLDN18, claudin 18.

Figure S1. CONSORT diagrams of patient populations in SPOTLIGHT, GLOW, ILUSTRO, and the phase 1 study.

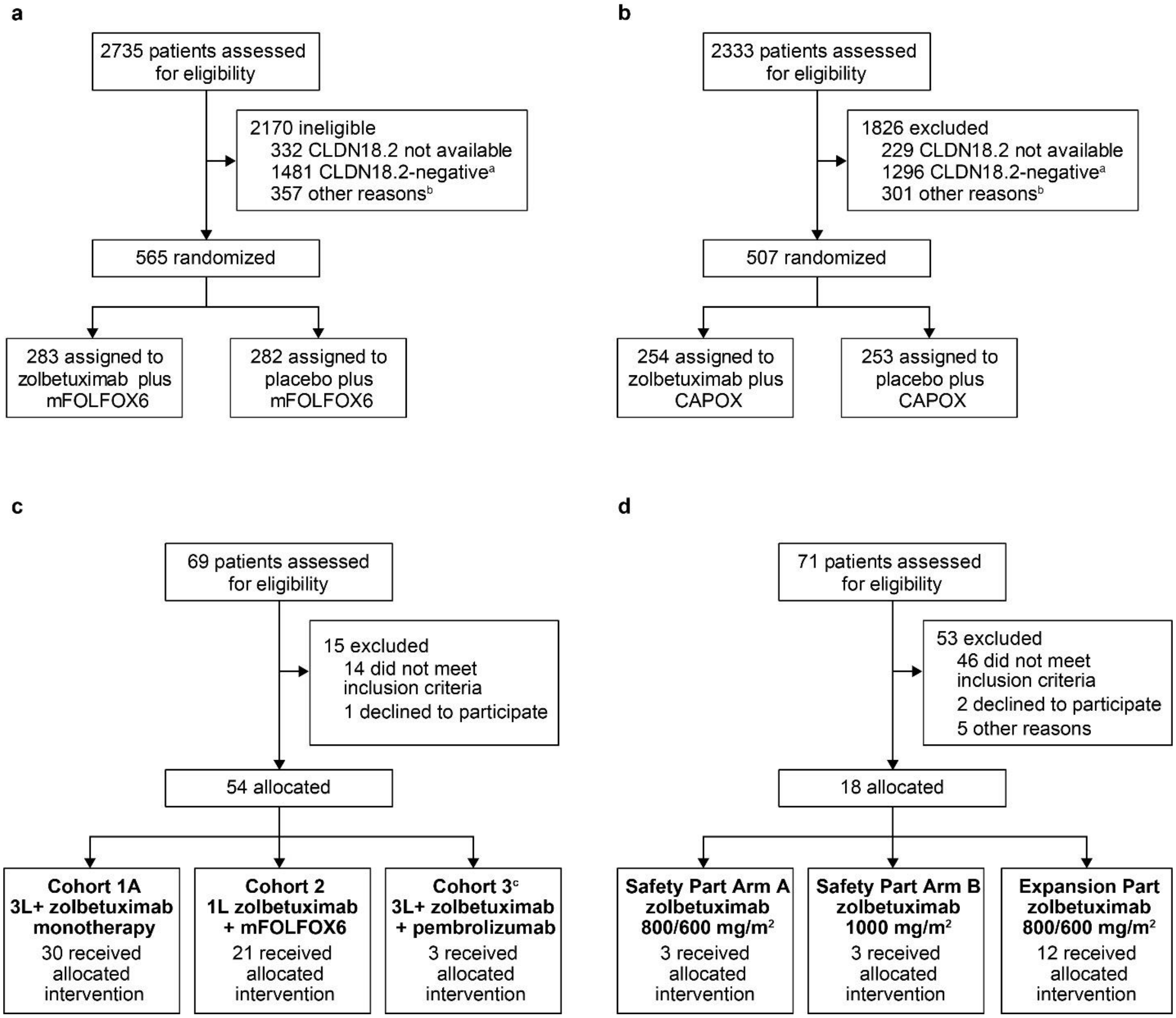

A, screening and random assignment of patients in SPOTLIGHT. **B,** screening and random assignment of patients in GLOW. **C,** screening and allocation of patients in ILUSTRO. **D,** screening and allocation of patients in the phase 1 study.

[a]CLDN18.2 positivity was defined as ≥75% of tumor cells demonstrating moderate-to-strong membranous CLDN18 staining using the VENTANA CLDN18 (43-14A) RxDx Assay (for Investigational Use Only; VMSI/Roche).

[b]"Other" represents patients with CLDN18.2-positive tumors who failed screening for other reasons including withdrawal by patient, laboratory findings, HER2-expression status, and Eastern Cooperative Oncology Group performance-status score.

[c]Tumor samples from patients allocated to cohort 3 of ILUSTRO were not analyzed in this study.

CAPOX, capecitabine and oxaliplatin regimen; CLDN18, claudin 18; CLDN18.2, claudin 18 isoform 2; CONSORT, Consolidated Standards of Reporting Trials; HER2, human epidermal growth factor receptor 2; mFOLFOX6, modified folinic acid, fluorouracil, and oxaliplatin regimen.

Figure S2. Prevalence of CLDN18.2 positivity[a] among all screened patients with HER2-negative tumors in SPOTLIGHT and GLOW by region.

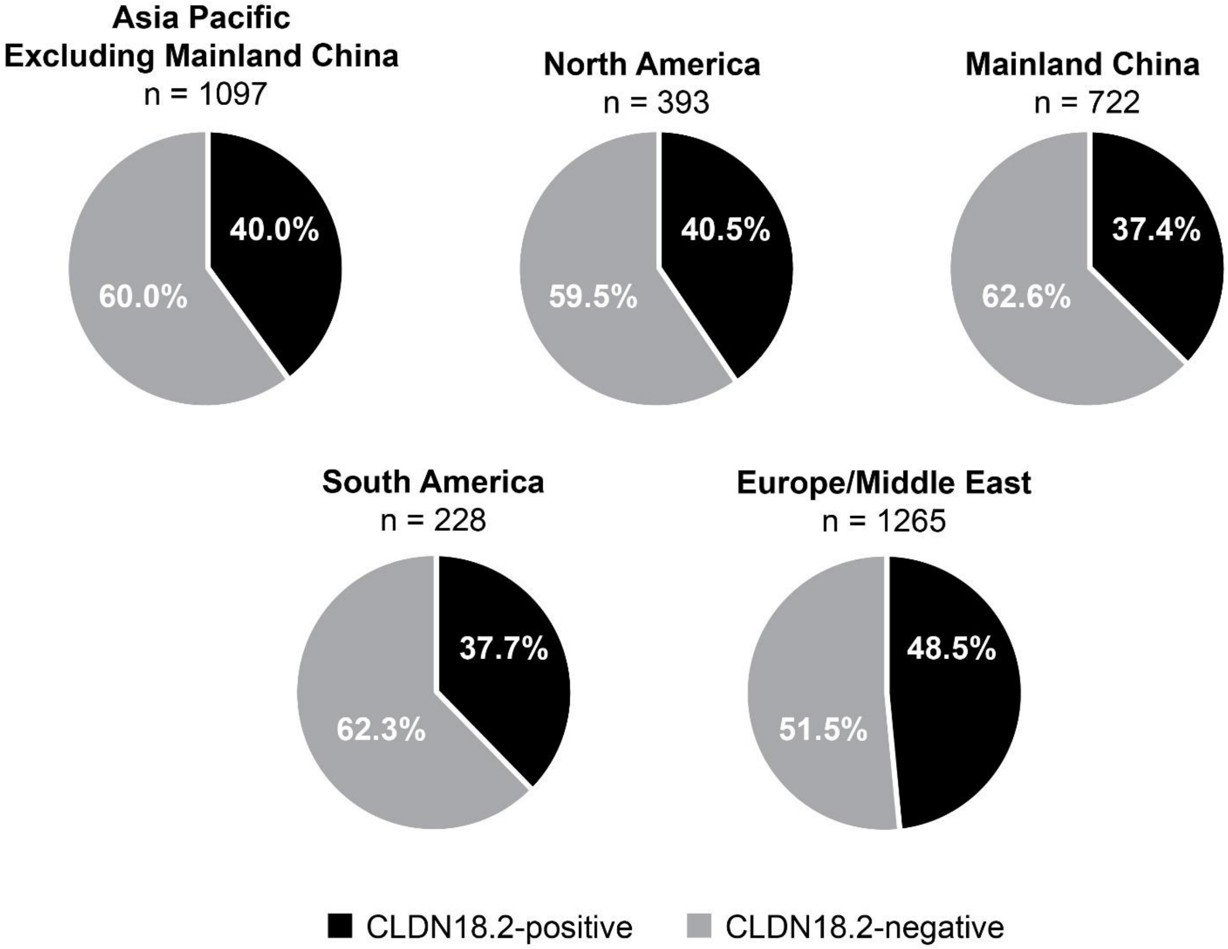

[a]CLDN18.2 positivity was defined as ≥75% of tumor cells demonstrating moderate-to-strong membranous CLDN18 staining using the VENTANA CLDN18 (43-14A) RxDx Assay (for Investigational Use Only; VMSI/Roche).

CLDN18, claudin 18; CLDN18.2, claudin 18 isoform 2; HER2, human epidermal growth factor receptor.

Supplementary references

1. Kubota Y, Kawazoe A, Mishima S, Nakamura Y, Kotani D, Kuboki Y, et al. Comprehensive clinical and molecular characterization of claudin 18.2 expression in advanced gastric or gastroesophageal junction cancer. ESMO Open. 2023;8(1):100762. doi: 10.1016/j.esmoop.2022.100762.
2. Pellino A, Brignola S, Riello E, Niero M, Murgioni S, Guido M, et al. Association of CLDN18 protein expression with clinicopathological features and prognosis in advanced gastric and gastroesophageal junction adenocarcinomas. J Pers Med. 2021;11(11). doi: 10.3390/jpm11111095.
3. Janjigian YY, Shitara K, Moehler M, Garrido M, Salman P, Shen L, et al. First-line nivolumab plus chemotherapy versus chemotherapy alone for advanced gastric, gastro-oesophageal junction, and oesophageal adenocarcinoma (CheckMate 649): a randomised, open-label, phase 3 trial. Lancet. 2021;398(10294):27-40. doi: 10.1016/S0140-6736(21)00797-2.
4. Sung H, Ferlay J, Siegel RL, Laversanne M, Soerjomataram I, Jemal A, et al. Global cancer statistics 2020: GLOBOCAN estimates of incidence and mortality worldwide for 36 cancers in 185 countries. CA Cancer J Clin. 2021;71(3):209-49. doi: 10.3322/caac.21660.
5. Rha SY, Oh DY, Yanez P, Bai Y, Ryu MH, Lee J, et al. Pembrolizumab plus chemotherapy versus placebo plus chemotherapy for HER2-negative advanced gastric cancer (KEYNOTE-859): a multicentre, randomised, double-blind, phase 3 trial. Lancet Oncol. 2023;24(11):1181-95. doi: 10.1016/S1470-2045(23)00515-6.
6. Shitara K, Lordick F, Bang YJ, Enzinger P, Ilson D, Shah MA, et al. Zolbetuximab plus mFOLFOX6 in patients with CLDN18.2-positive, HER2-negative, untreated, locally advanced unresectable or metastatic gastric or gastro-oesophageal junction adenocarcinoma (SPOTLIGHT): a multicentre, randomised, double-blind, phase 3 trial. Lancet. 2023;401(10389):1655-68. doi: 10.1016/S0140-6736(23)00620-7.
7. Shah MA, Shitara K, Ajani JA, Bang YJ, Enzinger P, Ilson D, et al. Zolbetuximab plus CAPOX in CLDN18.2-positive gastric or gastroesophageal junction adenocarcinoma: the randomized, phase 3 GLOW trial. Nat Med. 2023. doi: 10.1038/s41591-023-02465-7.
